# siRNA Nanoparticle Delivery Strategies and Clinical Trial Advances in Tumor Therapy

**DOI:** 10.3390/ijms27073032

**Published:** 2026-03-26

**Authors:** Pingjie Wang, Jing Gong, Yilin Xu, Xinhua Xia

**Affiliations:** School of Pharmacy, Hunan University of Chinese Medicine, Changsha 410208, China; wpj13237482039@163.com (P.W.); 15243786189@163.com (J.G.); xyl313798907@163.com (Y.X.)

**Keywords:** RNA interference, siRNA, siRNA drug nanoparticles, tumor therapy

## Abstract

siRNA, as a precise, specific, and highly effective gene-silencing therapy, has been extensively studied. Before reaching tumor cell targets, siRNA formulations must overcome multiple extracellular barriers, including clearance from the bloodstream, membrane impermeability, capture by the mononuclear phagocyte system (MPS), rapid renal excretion, endosomal escape, and precise recognition of target cells. These challenges limit siRNA’s clinical application. Consequently, various modifications have been applied to siRNA to enhance transfection efficiency, while researchers continue to pursue improved siRNA-targeting delivery systems. Nanotechnology offers a rational technical approach to address siRNA delivery. Nanoparticles can increase transfection efficiency while exhibiting lower cytotoxicity and reduced off-target effects. Various matrices have been employed to construct nanoparticles for targeted therapeutic delivery. This review briefly discusses siRNA nanoparticle delivery strategies, illustrates examples of various siRNA nanodelivery systems, such as lipid nanoparticles, polymeric siRNA nanoparticles, inorganic nanoparticles, hybrid nanoparticles, and conjugate-siRNA delivery systems, and introduces clinical trials of siRNA-loaded nanoparticles for cancer treatment, which can provide valuable references for further research and clinical application of siRNA nanoparticle delivery systems.

## 1. Introduction

**Figure 1:**
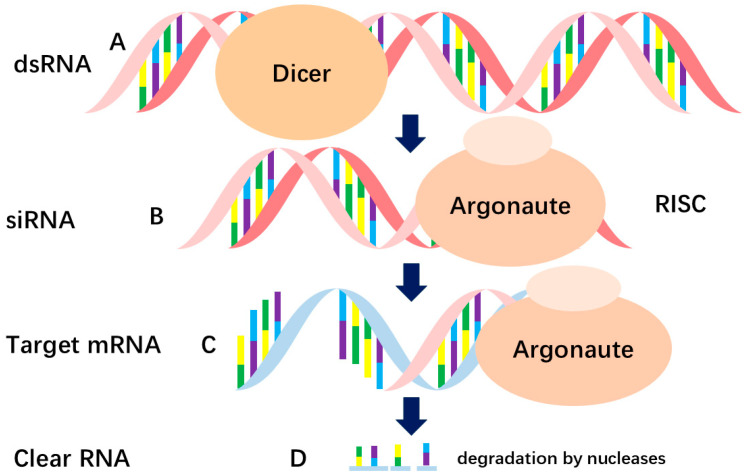
RNA Interference Process. A–B. dsRNA is cleaved by Dicer into siRNA, which is then unwound. The guide strand is assembled into the RNA-induced silencing complex (RISC), containing Argonaute proteins (catalytic components) for cleavage. B–C. The antisense strand pairs perfectly with the target mRNA as the guide strand. C–D. mRNA is cleaved and degraded.

RNA interference (RNAi) is a highly conserved post-transcriptional gene-silencing mechanism in eukaryotes [1]. In this process, exogenously introduced or endogenously produced double-stranded RNA (dsRNA) is cleaved by Dicer into small interfering RNA (siRNA) fragments of 21–23 nucleotides in length. Subsequently, siRNA is loaded into the RNA-induced silencing complex (RISC), where the antisense strand acts as the guide strand. It forms a fully complementary base-pairing with the target mRNA, mediating its specific cleavage and degradation, thereby silencing the expression of the target gene at the translational level [2] (Figure 1). Epigenetically relevant ncRNAs include small interfering RNA (siRNA), microRNA (miRNA), piwi-interacting RNA (piRNA), and long intergenic noncoding RNA (lincRNA) [3,4]. Among these, siRNA has become a primary focus for researchers and the pharmaceutical industry due to its high specificity, efficiency, and ease of design.

Some studies suggest that cancer is associated with single-gene or multi-gene defects, and focus on developing corresponding genetic materials to treat various types of cancer. In 2001, Elbashir et al. first successfully achieved siRNA-mediated gene silencing in mammalian cells [5]. Since then, siRNA therapy has demonstrated significant potential in multiple disease models, particularly in cancer treatment. Research confirms that silencing key oncogenes (e.g., *KRAS*, *MYC*, *BCL-2*), angiogenesis factors (e.g., *VEGF*), anti-apoptotic genes, or drug resistance-associated genes via siRNA effectively inhibits tumor growth and angiogenesis while enhancing chemotherapy sensitivity [6]. However, siRNA also suffers from drawbacks, including poor stability, a short half-life, and a lack of targeting. Furthermore, siRNA molecules are relatively large and carry a strong negative charge, making passive diffusion through the similarly negatively charged phospholipid bilayer of the cell membrane difficult [7]. Certain siRNA sequences, particularly those containing specific motifs (e.g., 5′-UGU-3′ or GU-rich sequences), may be recognized by Toll-like receptors (TLR3, TLR7, TLR8), activating innate immune responses. This leads to the release of type I interferons and inflammatory mediators, causing side effects such as fever and chills [8].

Nanotechnology offers an ideal platform for addressing this challenge. Nanoparticles exhibit excellent biocompatibility and biodegradability. When conjugated with siRNA, they significantly enhance the stability and efficacy of siRNA within the body. On one hand, nanocarriers encapsulate siRNA within their hydrophobic cores or compress it through electrostatic interactions, forming a physical barrier that effectively prevents degradation by nucleases and substantially prolongs its half-life in the bloodstream [9]. On the other hand, nanoparticles typically carry positive charges or undergo surface modifications, enabling enhanced interaction with negatively charged cell membranes through electrostatic interactions or ligand-receptor-mediated active targeting, thereby improving the cellular endocytosis efficiency of siRNA [10]. However, excess cation density has been shown in several studies to be strongly associated with safety issues such as cytotoxicity, complement activation, and nonspecific protein adsorption. Furthermore, the tunability of nanocarriers allows for the delivery of other therapeutic agents while ensuring minimal off-target effects and immunogenicity, enabling synergistic gene therapy with chemotherapy, phototherapy, and immunotherapy [11].

Significant advancements have been made in siRNA technology, including the development of stable chemically modified siRNA groups and various novel nanoparticle delivery systems. The loading methods for siRNA nanoparticles primarily include electrostatic complexation, hydrophobic entrapment, cavity encapsulation, and covalent conjugation. Among these, cationic carriers achieving high-efficiency loading through electrostatic interactions represent the most commonly employed strategy [12]. siRNA loading efficiency exhibits significant particle type dependence: Lipid nanoparticles (LNPs), cationic polymers, and lipid–polymer hybrid particles achieve high loading efficiencies of 80–95% through charge interactions; inorganic nanoparticles rely on surface modifications and pore structures, typically achieving loading efficiencies of 60–90%; bionic nanocarriers achieve gentle encapsulation via cell membranes or protein scaffolds, with efficiency significantly influenced by preparation techniques [13]. siRNA itself possesses high hydrophilicity and a negative charge, making it difficult to directly enter hydrophobic carriers into the nucleus. Therefore, it is typically loaded as a nucleic acid–cationic complex, where its hydrophilicity affects particle self-assembly, stability, and release rate. To determine the exact amount of siRNA released at the designated site, we need to conduct a release analysis. This analysis primarily measures cumulative release quantities through methods such as in vitro dialysis and centrifugal sampling, including release kinetics such as burst release effects, pH responsiveness, and time dependence [14]. Additionally, to achieve precise and controllable release, multiple external and endogenous stimuli are employed to regulate siRNA delivery: endogenous stimuli include tumor acidic pH, high glutathione concentrations, and enzyme responses; exogenous stimuli encompass near-infrared light, temperature, magnetic fields, and ultrasound. These can precisely trigger carrier disassembly and siRNA release in spatiotemporal dimensions, enhancing delivery efficiency while reducing toxic side effects. Meiling Zhang et al. designed a novel nanoparticle, PFPR (PDA-Fe-PEI-RGD), utilizing anti-*PD-1/PD-L1* immunotherapy targeting *EGFR*, demonstrating significant antitumor effects in treating metastatic non-small cell lung cancer (NSCLC) [15]. Another study developed and evaluated a cRGD-modified, pH-sensitive liposome system for the targeted co-delivery of docetaxel (DTX) and *ABCG2*-targeting siRNA (si-*ABCG2*). The synthesized nanoparticles (DTX/siRNA/cRGD-PLPs) exhibited favorable systemic tolerance in the MDA-MB-231 xenograft mouse model and significantly inhibited tumor growth. These effects were associated with reduced cell proliferation (Ki-67) and increased apoptosis (Caspase-3) in tumor tissues, demonstrating significant potential for improving the treatment of triple-negative breast cancer [16]. Furthermore, multiple siRNA nanomedicines for cancer therapy have advanced to clinical trial phases. The siRNA formulation Atu027 targeting protein kinase N3 (*PKN3*), studied by Aleku in 2008, entered Phase I clinical trials when combined with gemcitabine for advanced pancreatic cancer [17]. Another siRNA nanoparticle (DCR-*HIF2α*) targeting hypoxia-inducible factor-1α (*HIF-1α*) is currently being evaluated in patients with advanced solid tumors [18]. Furthermore, the U.S. Food and Drug Administration (FDA) has approved five siRNA-based therapeutics: patisiran, givosiran, lumasiran, inclisiran, and vutrisiran for treating TTR-mediated amyloidosis, primary hyperoxemia, acute porphyria, hypercholesterolemia, and amyloidosis, respectively [19]. This article briefly discusses delivery strategies for siRNA nanoparticle drugs, illustrating examples of lipid, polymeric siRNA nanoparticles, inorganic nanoparticles, hybrid nanoparticles, and conjugate-siRNA delivery systems, while also introducing clinical trials of siRNA-mediated nanoparticles for cancer treatment.

## 2. Delivery Barriers for siRNA Nanoparticles

**Figure 2:**
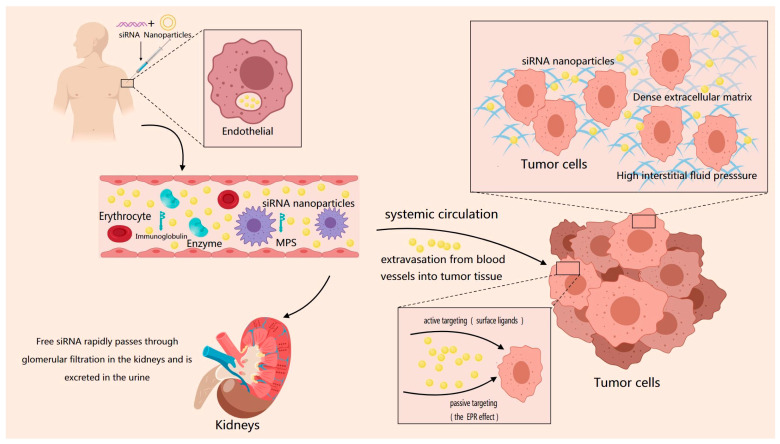
The delivery difficulties in siRNA formulations before reaching the tumor cell membrane. After siRNA is loaded onto nanoparticles and administered intravenously into the bloodstream, it encounters multiple barriers, including blood clearance, RES cell phagocytosis, nuclease degradation, physicochemical modifications, renal filtration, and other barriers. It then traverses the vascular wall via the EPR effect, relying on vascular permeability, and subsequently diffuses within the tumor stroma. Confronted by high interstitial pressure, cellular crowding, and dense ECM, it ultimately reaches target cells through the specificity of targeting ligands.

**Figure 3:**
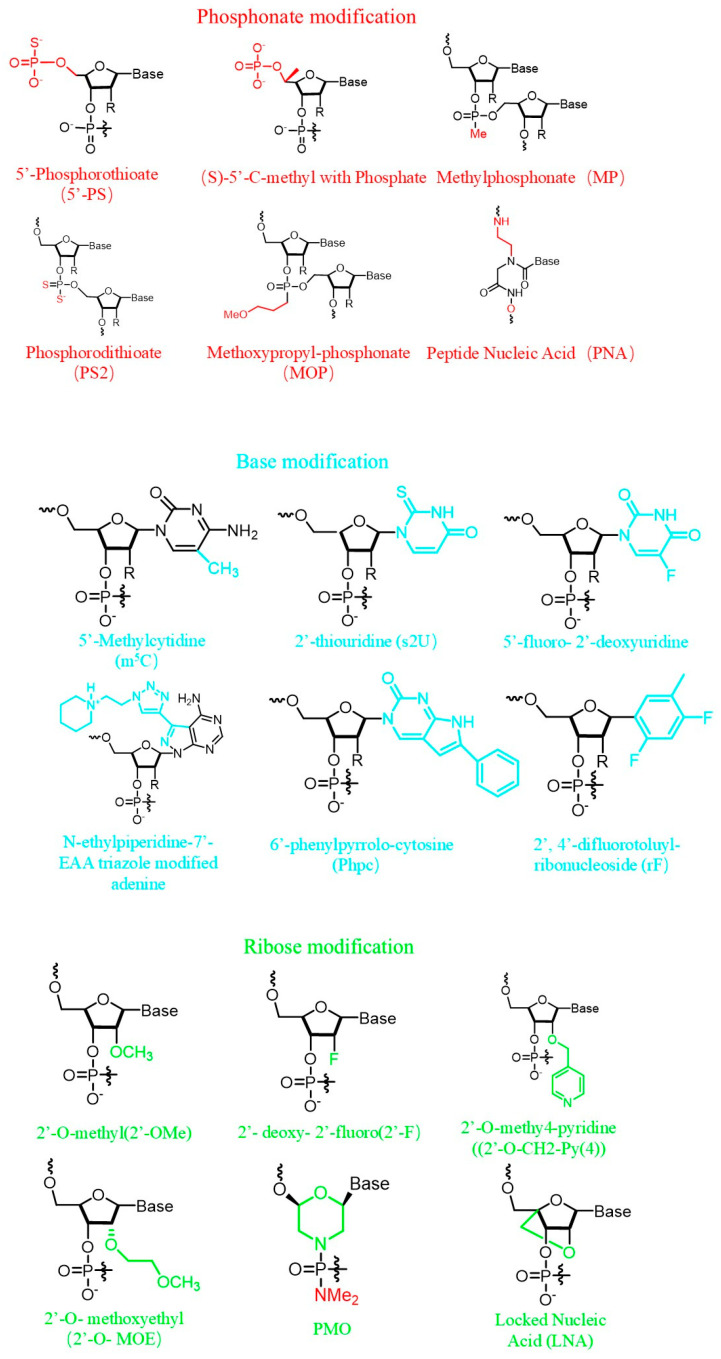
Schematic diagram of partial chemical modification structures of siRNA.

The siRNA formulations must overcome multiple extracellular barriers before reaching tumor cell membranes, including clearance from the bloodstream, crossing vascular walls, permeating tumor stroma, and achieving precise target cell recognition [20] (Figure 2). The first barrier is vascular clearance. When siRNA formulations enter the body via intravenous injection, exposed siRNA is rapidly degraded within minutes by ubiquitous nucleases (plasma nucleases and lysosomal nucleases) in the bloodstream, reducing its bioavailability [13]. Subsequently, they undergo renal clearance. Small-molecular-weight (~13 kDa) and hydrophilic siRNA, if not complexed, are rapidly excreted via glomerular filtration [21]. This process is further complicated by recognition and clearance by the immune system. siRNA carriers typically carry a positive charge, while proteins carry a negative charge under physiological conditions. The strong electrostatic interaction between the siRNA carriers and proteins leads to a large number of negatively charged plasma proteins, such as immunoglobulin IgG/IgM, which are nonspecifically adsorbed and enriched on the surface of the carrier to form a protein corona. This protein arrangement mimics the structure of the natural immune complex and activates the complement classical pathway. At the same time, the cationic surface of siRNA interferes with the normal regulation of complement inhibitory proteins, leading to activation of the complement bypass pathway [22]. Blood proteins (e.g., immunoglobulins, complement) adsorb onto nanoparticle surfaces (corynophores), leading to recognition and phagocytosis by macrophages within the mononuclear phagocyte system (MPS) [23]. This represents the primary route for nanoparticle clearance from the bloodstream. To avoid blood clearance and reduce immune activation, the internal nucleotide structure of siRNA itself can be chemically modified. The main methods include phosphorothioate modification, ribosylation, and base modification (Figure 3). Encapsulating nanoparticles with hydrophilic molecules like polyethylene glycol (PEG) and using ionizable lipid nanoparticles (LNPs) enables siRNA delivery carriers to evade recognition by the immune system and associated phagocytes [24]. Encapsulating siRNA within the hydrophobic core of LNPs or tightly compressing it via electrostatic interactions creates a physical barrier that completely isolates nucleic acids from nucleases. Incorporating PEG chains onto the LNP surface forms a hydrophilic “cloud,” reducing serum protein adsorption (anti-cating) and thereby avoiding rapid recognition and clearance by hepatic macrophages, prolonging circulation time.

Patisiran is the first approved siRNA drug, perfectly embodying the aforementioned strategy [25]. Patisiran utilizes a precisely engineered LNP system composed of ionizable lipids, cholesterol, auxiliary phospholipids, and PEG lipids to achieve liver targeting via apolipoprotein E (ApoE)-mediated uptake by hepatocytes. Researchers have also designed systems where a peptide chain is attached to the PEG end. This peptide chain can be cleaved in the microenvironment of the tumor stroma, where matrix metalloproteinases (MMPs) are overexpressed. Before PEG detachment, the nanoparticle exhibits long circulation; after detachment, the exposed targeting ligand or positive surface promotes cellular uptake [26].

The second barrier is vascular extravasation, where nanoparticles must traverse the vascular wall from the circulatory system into tumor tissue. In normal tissues, the vascular endothelium is tightly packed, making extravasation difficult. In tumor tissues, however, while vascular abnormalities and leakage create enhanced permeability and retention effects (EPR), they exhibit high heterogeneity [27]. First, not all tumor regions possess high permeability. The uneven distribution of vascular leakage zones leads to non-uniform drug distribution. The absence of functional lymphatics in tumor tissue impedes interstitial fluid drainage, creating interstitial hydrostatic pressure that counteracts nanoparticle diffusion from blood vessels into tumor tissue. Building on this, optimizing nanoparticle size, charge, and shape can harness and enhance the EPR effect to improve extravasation. Particles within the 10–100 nm range (particularly 30–50 nm) are less susceptible to renal filtration while readily traversing tumor vascular gaps [28]. Temporarily reducing matrix density and interstitial hydrostatic pressure can be achieved through co-delivery or pre-treatment with ECM-degrading enzymes such as hyaluronidase or collagenase [29]. PEGylated recombinant human hyaluronidase (PEGPH20), developed by Provenzano, P. P., effectively degrades hyaluronic acid-rich tumors, reduces interstitial hydraulic pressure, and enhances subsequent chemotherapy drug delivery [30].

The third obstacle is the specific recognition between nanoparticles and target cells. Nanoparticles must be internalized by the correct cell type. Nanoparticles lacking active targeting ligands primarily rely on nonspecific endocytosis, which is inefficient and lacks selectivity. One approach to overcome this limitation is to program nanocarriers to actively bind specific cells after release [31], targeting specific organs and tissues through surface ligand modification. Currently, antibodies, peptides, and aptamers have been reported as targeting ligands that promote the accumulation of siRNA-containing nanoparticles at specific sites. Table 1 presents the advantages and limitations of antibody, peptide, and aptamer modification methods. Givosiran, an FDA-approved siRNA, binds to GalNAc. GalNAc targets the asparaginase receptor (*ASGPR*), enabling drug enrichment in the liver.

Moreover, a novel “camouflage” strategy—cell membrane-coated nanoparticles—has demonstrated tremendous potential in the field of tumor therapy. By encapsulating the nanocarrier within a cell membrane, it enables active targeting of tumor sites. Primarily based on “membrane origin (cell type)” and paired with corresponding target-locking mechanisms, specific nanocarriers and anticancer drugs are encapsulated to achieve therapeutic effects in various cancer models. Based on the source of the membranes, they can be classified into red blood cell membranes, white blood cell membranes, platelet membranes, tumor cell membranes, and others. Biomimetic particles coated with different types of cell membranes exhibit distinct functionalities. Nanoparticles coated with tumor cell membranes possess homogenous targeting capabilities, enhancing drug accumulation in tumor tissues while reducing elimination by the immune system [35]. Sun H et al. utilized tumor cells to encapsulate polymeric nanoparticles (PNs) + paclitaxel (PTX), demonstrating that this nanoparticle can extend drug circulation time and inhibit primary lung metastasis in the 4T1 breast cancer model [36]; Table 2 lists several cell membrane-coated nanoparticles and their associated studies. Moreover, numerous types of stem cells, such as bone marrow-derived mesenchymal stem cells (MSCs) and neural stem cells (NSCs), have been demonstrated to possess the ability to specifically target tumors [37]. Engineered stem cells can secrete molecules such as tumor necrosis factor-related apoptosis-inducing ligand (TRAIL), interferon-β (*IFN-β*), and IL-12/18 factors in tumor tissue regions, thereby inhibiting tumor cell proliferation [38]. Mesenchymal stem cells primarily target tumor cells through cell-to-cell surface contact and chemotaxis. Their targeting mechanism is tumor-specific rather than species-specific, enabling different types of mesenchymal stem cells to target distinct tumor cell types through selective isolation. Furthermore, stem cells exhibit low immunogenicity, enabling the use of different mesenchymal stem cell membranes to surface-modify nanocarriers [39]. This approach facilitates the preparation of nanogel particles modified with stem cell membranes, enhancing the nanocarriers’ resistance to immune clearance, their ability to undergo prolonged circulation in the bloodstream, and their tumor-targeting recognition capabilities within the body [40]. IIgin Kimiz-Gebologlu established a THP-1 pre-monocyte cell line for exosome production, significantly enhancing exosome yield while shrinking tumor spheroids and reducing cell survival rates, demonstrating tumor suppression and antigen delivery capabilities [41].

After siRNA enters cells, the primary obstacles it faces are entrapment within endosomes and immune responses. Endosomes can be disrupted using endodermic dissolver agents, including polymers, small molecules, and peptides. Alternatively, the proton sponge effect can also be utilized, wherein chloride ions flow into the endosome, causing disruption of the endosomal membrane and release of its contents, such as representative drugs like Givosiran. Another protonation mechanism may also be exploited, whereby amino acids—particularly lysine and arginine—become protonated at low pH, leading to membrane disruption. Poly-L-arginine (PLA) can also disrupt endosomal membranes via the proton sponge effect, enabling efficient siRNA release into the cytoplasm [47]. Additionally, siRNA activates the innate immune system, promoting cytokine production in vivo. Certain GU-rich or specific motif siRNA sequences, or nanocarrier materials like cationic lipids/polymers, may activate the innate immune system. This primarily occurs via Toll-like receptors (TLRs) 3/7/8 or cytoplasmic sensors, leading to inflammatory cytokine release. This can cause side effects such as fever and chills and may accelerate carrier clearance [48].

## 3. siRNA Nanoparticle Delivery System

### 3.1. Lipid-Based Nanoparticle Delivery Systems

Lipid molecules exhibit amphiphilic properties, making them ideal carriers for encapsulating both hydrophobic and hydrophilic therapeutic drugs. Among these, liposomes composed of one or more phospholipid bilayers—such as phosphatidylcholine or phosphatidylethanolamine—have been extensively utilized in the treatment of liver diseases [49]. The first FDA-approved siRNA drug, Patisiran, utilizes a lipid-based delivery system [50]. Lipid delivery nanosystems rely on electrostatic complexation and hydrophobic core–shell assembly to load siRNA, achieving high and stable drug loading efficiencies typically ranging from 80% to 95%, with drug loading capacities generally between 5% and 12%. The release kinetics exhibit a typical biphasic pattern: a moderate burst release of 15–30% within the initial 24 h, followed by a slow sustained release phase where 50–75% is cumulatively released between 48 and 72 h. Release is rapidly triggered in the acidic environment (pH 5.0–6.0) of endosomes, significantly enhancing intracellular siRNA delivery efficiency [51]. Solid lipid nanoparticles (SLNs) consist of physiologically tolerated lipids, such as glycerol monostearate or stearic acid, while nanostructured lipid carriers (NLCs) contain both solid and liquid lipids, such as triacylglycerols and oleic acid. This structure enhances drug loading capacity, protects against enzymatic degradation, and exhibits controlled release kinetics, making it suitable for sustained delivery of poorly water-soluble drugs to liver tissue [52]. Lipid nanoparticles (LNPs) composed of ionizable cationic lipids (such as DLin-MC3-DMA), cholesterol, phospholipids, and PEG lipids have emerged in recent years as highly efficient carriers for nucleic acid therapeutics like siRNA and mRNA due to their endosomal escape capability and high-efficiency transfection capacity in hepatocytes [53]. Lipid-based nanoparticles represent one of the most extensively studied and clinically validated liver-targeted drug delivery platforms. Abdulrahman et al. developed a bivalent lipid-binding siRNA (L2-siRNA), targeting Spp1 in BAMs to alleviate neuroinflammation induced by Alzheimer’s disease (AD). L2-siRNA exhibits dose-dependent effects and can potently silence Spp1 expression. Following intracerebroventricular (ICV) injection, it distributes extensively throughout the central nervous system, preferentially targeting and internalizing BAM [54]. Tazawa et al. constructed LNPs encapsulating anti-type II transmembrane serine protease 4 siRNA. Using NUGC-3 cell-implanted gastric cancer models in nude mice, they evaluated the biodistribution and antitumor efficacy of the LNPs in vivo and in vitro. LNPs demonstrate enhanced accumulation within tumors, and the combination of LNPs with 5-FU significantly inhibits gastric cancer growth compared to 5-FU alone [55].

#### 3.1.1. Liposomes

Liposomes are spherical vesicles composed of one or more phospholipid bilayers, and they typically contain components such as phosphatidylcholine, cholesterol, and phosphatidylethanolamine. Due to their structural diversity and ability to encapsulate both hydrophilic and lipophilic drugs simultaneously, liposomes have been extensively studied for siRNA delivery [56]. PEG modification reduces protein adsorption, decreases MPS clearance, prolongs drug circulation, and promotes passive drug accumulation in diseased liver tissue through the enhanced permeability and retention (EPR) effect. Some studies have also employed chitosan and chitosan-based composites as carriers and coupled them with liposomes to enhance cellular uptake efficiency [57]. Liposomes enter cells mainly through membrane fusion or endocytosis. Induced by the acidic environment in endosomes, the lipid bilayer structure dissociates, triggering endosomal escape and the release of the loaded siRNA. Cationic liposomes encapsulate the ribonucleic acid by forming a lipid complex through an interaction with the negative charge of siRNA. Theoretically, cationic liposomes are considered to possess superior stability and efficiency. Recently, a novel study introduced the method of ethanol injection (MEI) for preparing anionic siRNA lipid complexes, demonstrating favorable cellular uptake and gene-silencing effects. First, a lipid–ethanol solution comprising one of four cationic lipids [1,2-dioleoyl-3-trimethylammonium-propane methyl sulfate salt (DOTAP), dimethyldioctadecylammonium bromide (DDAB), N-hexadecyl-N,N-dimethylhexadecan-1-aminium bromide (DC-1-16), or 11-((1,3-bis(dodecanoyloxy)-2-((dodecanoyloxy)methyl)propan-2-yl)amino)-N,N,N-trimethyl-11-oxoundecan-1-aminium bromide (TC-1-12)], DOPE, and PEG-Chol was combined with siRNA in PBS. Next, a lipid–ethanol solution of cholesteryl hemisuccinate (CHS) and DOPE was added. This mixture was then combined with a lipid–ethanol solution containing CHS and DOPE. During this process, the zeta potential of the lipid complex shifts from positive to negative. In MCF-7 and HeLa cells, the TC-1-12 complex achieved potent gene silencing while reducing interactions with red blood cells, confirming the efficacy of the two-step MEI method for preparing anionic siRNA lipid complexes [58]. Researchers constructed liposomes targeting *ApoB* peptides to encapsulate small interfering siRNA, targeting herpes simplex virus type 1 (HSV1). Following systemic intraperitoneal administration, preferential accumulation occurs in the trigeminal ganglion, but stability is poor. Particle aggregation is observed after one month of storage at 4 °C, and rapid fluorescence quenching occurs when labeling lipid membranes with various fluorescent probes [59]. Another research has utilized 2X3-DOPE as a structural framework to construct cationic liposomes, investigating the influence of structural characteristics and combination patterns of folic acid-conjugated targeting molecules (F13) with two shielding lipid conjugates (P1500, diP1500) on siRNA delivery efficiency both in vivo and in vitro. Research has found that the cyclic PEG–lipid conjugate diP1500, containing two hydrophobic anchoring groups, exhibits optimal performance. The F13/diP1500-conjugated liposome had a particle size of 126.0 ± 23.0 nm and maintained a strong positive charge of 21.6–30.5 mV across all N/P ratios, effectively circumventing the “polyethylene glycol dilemma”. F13/P1500 composite liposomes exhibited a particle size of 241.8 ± 65.7 nm and a potential of 3.9–9.1 mV. F13-modified liposomes promote siRNA cellular accumulation via receptor-mediated pathways. DOPE-PEG2000/diP1500 liposomes reached a peak plasma concentration (1.84 ± 0.01 pmol/mL) at 15 min post-administration, demonstrating significant circulatory advantages. This study represents the first direct comparison, in vivo, of lipid conjugates with identical PEG chain lengths but differing numbers of anchoring groups, revealing delivery efficacy distinctions arising from membrane structural variations. These findings provide critical reference points for the rational design of targeted liposomes [60].

#### 3.1.2. Solid Lipid Nanoparticles (SLNs) and Nanostructured Lipid Carriers (NLCs)

SLNs are composed of biocompatible solid lipids such as glycerol monostearate and stearic acid. It exists as a solid crystalline phase at both ambient and physiological temperatures, enabling controlled drug release, resistance to enzymatic degradation, and enhanced bioavailability [61]. Multiple methods can be employed to develop SLNs, including solvent evaporation, spray drying, supercritical fluid extraction of emulsions, and ultrasonication [62]. NLC, as a second-generation lipid nanocarrier, is an improved version of SLNs. The NLC combines solid lipids, such as glycerol ethers, with liquid lipids (oleic acid or medium-chain triglycerides) to form a partially amorphous lipid matrix, enhancing drug loading capacity and preventing drug leakage. NLCs offer multiple advantages, including enhanced physical stability, controllable particle size, and superior capture of hydrophilic and lipophilic drugs. NLCs also present several barriers, including matrix-associated cytotoxicity, concentration, and the irritating effects of certain surfactants. Jagdale et al. [63] constructed N-acetyl-d-glucosamine-modified paclitaxel-loaded solid lipid nanoparticles (GLcNAc-PTX-SLN) to target the *GLUT1* transporter highly expressed in ovarian cancer cells and inhibit tumor progression. The formulation exhibits a hydrodynamic diameter of 232 ± 3.21 nm and a polydispersity index of 0.210 ± 0.16. Cumulative drug release rates at pH 6.5 and pH 7.4 after 24 h were 80.54 ± 2.35% and 47.91 ± 1.83%, respectively, with a hemolysis rate below 5%. Cell experiments confirmed that it induced late-stage apoptosis in 95.31% of cells, demonstrating outstanding antitumor activity [63]. Rajoriya et al. [64] prepared folate-conjugated nano-lipid constructs (F-NLCs) targeting lung squamous carcinoma, with a particle size of 231.3 ± 2.3 nm and a zeta potential of 10.27 ± 0.2 mV. The encapsulation efficiency and drug loading capacity were 82.42 ± 1.2% and 24.2 ± 1.3%, respectively. In vitro drug release studies showed that 94.21% of the drug was released from the F-NLC formulation at pH 4.0 after 16 h, while 88.92% was released at pH 6.4 after 16 h. In vitro cytotoxicity studies demonstrated that F-NLCs exhibited higher cytotoxicity toward carcinoma cells, with GI_50_ values of 5.84 (molar concentration) and LC_50_ values of 48.9 µg/mL, proving that F-NLCs represent a platform for improving drug delivery efficiency and optimizing anticancer therapy [65]. Surface-modified lipid nanocarriers enhance targeting capabilities, offering diverse formulation strategies for tumor-targeted therapy.

#### 3.1.3. Lipid Nanoparticles (LNPs)

LNPs have revolutionized the field of nucleic acid therapeutics by providing a clinically viable platform for the safe and efficient delivery of RNA molecules. The fundamental principle underlying LNP formation is self-assembly, an intrinsic characteristic of intelligent nanosystems. Due to the low toxicity and biodegradability of LNPs, various lipids with distinct chemical properties have been explored for siRNA delivery. However, some studies have also indicated that LNPs may be associated with inducing inflammatory responses [66]. Following administration of certain LNP vaccines, human subjects have reported symptoms such as pain at the injection site, headache, and fever. Research has identified neutrophils as key effector cells in LNP-induced inflammation [67]. The development of 17,18-epoxyethyl ethanoic acid (17,18-EpETE) significantly reduces local swelling and infiltration of immune cells at the injection site. This anti-inflammatory effect is mediated by G protein-coupled receptor 40 (GPR40) and does not impair antibody generation induced by LNP vaccines. This demonstrates that 17,18-EpETE holds potential as an adjuvant, mitigating inflammatory side effects without compromising therapeutic efficacy. Lipids can enhance siRNA transfection efficiency through two chemical modifications: the first approach involves introducing multiple unsaturated alkyl chains to modify the lipid, causing it to adopt an overall “cone” conformation in acidic environments, which promotes the formation of non-bilayer phases. The second approach involves improving the initial fusion between lipid nanoparticles and cell membranes through the protonation of the diethylaminopropyl group, though this method is only effective for LNPs [68]. Sang M Lee et al. selected ionizable lipids with high mRNA in vitro delivery efficacy from 465 lipids, evaluating their in vivo activity and structure–activity relationships. They evaluated 42 lipids to study how the chemical alterations in their core internal carbon chains affect the therapeutic efficacy of LNPs. They found that pKa and buffering capacity are valuable for predicting lipid nanoparticle delivery to the liver in vivo, and extended the acceptable LNP pKa range to 6.2–7.4, providing new reference points for rational design of ionizable lipids for LNP delivery [69]. Another study has also investigated the effects of PEGylation on the biodistribution and gene silencing of siRNA–lipid nanoparticle complexes. They observed that a small yet sufficient amount of 3-N-[(ω-methoxypoly(ethylene glycol)2000)carbamoyl]-1,2-dimethylaminooxypropylamine (PEG-C-DMA) in LNPs plays a crucial role in achieving efficient in vivo silencing of the *FVII* gene. It was also found that PEGylation did not alter the distribution of LNPs in the liver, but it did change the efficacy of gene silencing, possibly by reducing endosomal degradation [70]. Matsuo-Tani et al. [71] established a modular Fc-binding peptide (FcBP)-mediated strategy for *PD-L1*-targeted *VEGF*-siRNA delivery in the treatment of glioblastoma (GBM). This targeted LNP complex reduced tumor volume by 65%, significantly suppressed bioluminescence signals, and did not cause weight loss. The anti-*PD-L1*–FcBP–LNP group exhibited a 63% reduction in final tumor weight (656.9 ± 125.4 mg), whereas the *VEGF*-siRNA group recorded 1794.1 ± 103.7 mg. Evidence shows that FcBP-modified LNPs retain antibody-directed and binding activity, enabling rapid functionalization of targeting antibodies. *PD-L1*–FcBP–LNP demonstrated potent and selective antitumor effects in GBM mouse models, exhibiting translational potential for treating GBM [71].

### 3.2. Polymeric Nanocarriers

Polymeric nanoparticles represent the most commonly employed delivery methods in targeted therapy. Polymeric nanocarriers encapsulate siRNA through electrostatic complexation, hydrophobic self-assembly, or micelle encapsulation, typically achieving loading efficiencies of 65–90%. These efficiencies are significantly influenced by the nitrogen-to-phosphorus ratio (N/P), polymer charge density, and preparation methodology. Release kinetics are primarily driven by polymer swelling, degradation, or chemical bond cleavage, exhibiting slow and sustained characteristics: approximately 20–40% is released within 24 h, with cumulative release typically reaching 50–85% by 72 h. Stimuli such as pH, GSH, or enzymes can trigger accelerated release, but this often results in delayed release and incomplete delivery, with total release rates frequently falling below 90%. Formaldehyde, alginate, cellulose, gelatin, and hyaluronic acid are widely used natural polymer nanomaterials, while synthetic polymer carriers include polyvinyl alcohol, polycaprolactone, PEI, PLGA, and polylactic acid [72]. Among these polymers, PEI has been the most extensively studied, primarily due to its strong buffering capacity (5.1~7.4), excellent binding affinity for genetic material, and significant transfection efficiency [73].

#### 3.2.1. Polymer Micelles

Polymer siRNA micelles are classified into two types based on their structure: (1) micelles formed by direct binding of siRNA to degradable or non-degradable PEG chains, or by further condensation using siRNA condensates to form micellar structures; (2) amphiphilic block copolymers containing multiple ions or lipid segments form complexes with siRNA, followed by the aggregation of these block copolymer–siRNA complexes into polymeric micelles [74]. Polymer nanoparticles are predominantly positively charged and bind nucleic acids through electrostatic interactions. The binding strength is influenced by molecular weight, charge density, and pH. Polymer micelles hold promise as superior options for enhancing anticancer drug delivery by leveraging surface-conjugated targeting compounds to boost therapeutic efficacy, provide synergistic drug effects, and prolong drug release duration [75]. Ziyu Zhou et al. creatively designed a potential ionizable polymer micelle (IPM) siRNA delivery system by combining three ionizable oligomers (IOs) with poly(lactic acid-polyethylene glycol) (PLA-PEG) to form IPMs encapsulating nucleic acid drugs. IPMs mitigate the accelerated blood clearance (ABC) effect, generate fewer PEG antibodies than LNPs, and exhibit significantly lower apolipoprotein adsorption in vivo compared to LNPs. They further introduced the targeting molecule *FAP*i to silence *FAP* overexpression, thereby achieving treatment of liver fibrosis. In vitro experiments demonstrated that *FAP*i-IPM exhibited approximately 20% higher uptake rates in activated hepatic stellate cells compared to IPM, with a dose-dependent effect observed. The RNAi efficiency targeting *HMGB1* and *HSP47* was increased to 55.39 ± 1.22% and 42.46 ± 3.49%, respectively. In vivo, *FAP*i-IPM exhibits significant targeting toward hepatic stellate cells in mice with liver fibrosis. Its phagocytic activity is lower than that of IPM, thereby more effectively knocking down *HMGB1* and *HSP47*, reducing ALT, AST, and TNF-α levels, and effectively alleviating liver fibrosis [76]. Currently, various polymeric micelles have been employed for siRNA delivery (Table 3).

#### 3.2.2. PEI-Modified Nanoparticles

PEI nanoparticles exhibit high transfection efficiency and superior endosome escape capability. PEI can be chemically modified with stearic acid (StA) prior to siRNA conjugation to enhance silencing activity. Yaozhen He et al. developed a carrier system composed of PEI modified with octa-arginine (R8) and utilized this modified PEI to fabricate dissolvable microneedles loaded with siRNA (siRNA@R8-PEI/DMNs). siRNA@R8-PEI/DMNs effectively penetrates Parafilm^®^ M film to a depth of approximately 380 μm. In vitro transfection assays demonstrated a transfection efficiency exceeding 80%. It effectively inhibited approximately 90% of A375 cell proliferation in a concentration-dependent manner. Additionally, the 24 h cell migration rate decreased to approximately 20%, with gene-silencing efficiency reaching up to about 74%. Its volume-based tumor suppression rate was approximately 67%, inducing tumor cell apoptosis and reducing *BRAF* expression. siRNA@R8-PEI/DMNs provide a promising transdermal siRNA delivery system for the treatment of cutaneous melanoma [84]. PEI exhibits certain cytotoxicity in vitro, but researchers have explored various strategies to address this issue. Fluorination has been demonstrated to be an effective method for reducing PEI cytotoxicity and enhancing the efficiency of siRNA delivery in vitro [85]. Lian X et al. employed PEI as the model cationic polymer to investigate the effects of fluorination on siRNA delivery in vitro and in vivo. Researchers fluorinated branched PEI with trifluoroacetic acid or perfluorobutyryl chloride in dichloromethane. They successfully mediated siRNA binding in the triple-negative breast cancer (TNBC) cell line, MDA-MB-231, by using fluorinated PEI carriers. Modifying PEI with neutral or anionic matrices has also been shown to reduce cytotoxic effects. Hydrophobic modifications have also been shown to improve siRNA delivery. Chen L et al. employed maltose, maltotriose, and hydrophobic n-octanal to jointly modify PEI, successfully synthesizing oligosaccharide-modified PEI (OM-PEI) and hydrophobic-modified OM-PEI (H-OM-PEI). Screening revealed that the PEI25k derivatives HC4 and HD4 exhibited optimal performance, achieving complete siRNA loading at a polymer-to-siRNA mass ratio of 4:1. Compared to unmodified PEI25k, they reduced protein adsorption by 50% and provided superior siRNA protection. Hydrophobic modification enables H-OM-PEIs to form compact nanoparticles, enhancing cellular uptake and achieving a 2.5-fold improvement in luciferase silencing efficacy compared to PEI25k in HeLa cells. In vivo experiments confirmed its significant enrichment in the liver and effective tumor targeting. This study demonstrates that oligosaccharide-alkyl dual modification of PEI balances charge regulation and delivery efficiency, mitigates inherent toxicity, and enhances siRNA stability [86]. Additionally, studies have determined the optimal spray-drying conditions for PEI-based nanoparticles containing large plasmid DNA or small siRNA. It was found that polyvinyl alcohol (PVA) is particularly suitable as an adjuvant, as it can maintain or even enhance transfection efficiency. This study provides a method for preparing a nucleic acid drug storage formulation that can be easily redissolved, demonstrating significant potential for direct pulmonary administration as a dry powder [87].

#### 3.2.3. PLGA-Modified Nanoparticles

PLGA is an FDA-approved copolymer that hydrolyzes into lactic acid and glycolic acid, both of which are naturally metabolized through the Krebs cycle. This makes it highly suitable for controlled and sustained drug release formulations [88]. PLGA nanoparticles have been studied for siRNA delivery, particularly for cancer treatment. However, PLGA nanoparticles face difficulties in escaping the endosome, which impedes the timely release of siRNA, thereby preventing its full activity from being demonstrated. Therefore, PLGA nanoparticles must undergo chemical modification to induce endosomal escape. Toruntay C et al. leveraged the high expression of the *MAPK6* gene in MCF-7 breast cancer cells to construct siRNA-loaded PLGA nanoparticles, exploring the potential of the *MAPK6* gene as a therapeutic target for breast cancer. Research has found that siMAPK6-PLGA-NPs efficiently suppress MAPK6 expression, significantly reducing the migration, proliferation, and colony-forming capacity of MCF-7 cells while enhancing apoptosis. This demonstrates the substantial potential of siRNA-loaded PLGA nanoparticles for breast cancer therapy [89]. Researchers have also developed folate-conjugated PLGA nanoparticles (FA-PLGA NPs) for folate receptor (FR)-targeted delivery of Toll-like receptor 4 small interfering RNA (TLR4 siRNA) to treat diabetic nephropathy (DN) [90]. Furthermore, PLGA nanoparticles can be modified using chitosan. Mohajeri S et al. utilized dual-purpose chitosan-polylactic acid-polyethylene glycol (PLA-PEG) nanoparticles to target glucose, thereby enhancing the delivery of paclitaxel (PTX) and small interfering RNA-fluorescein amide (siRNA-FAM) to cancer cells. This PLA-chitosan-PEG-glutamic acid copolymer exhibits low toxicity, and the delivery efficiency of siRNA-FAM is significantly higher than that of uncoated siRNA. The combination of chitosan and PLGA facilitates siRNA encapsulation, offering enhanced loading capacity, higher efficiency, and advanced delivery capabilities [91].

#### 3.2.4. Chitosan (CS) and Alginate

Chitosan (CS) is a naturally occurring cationic polysaccharide derived from deacetylated chitin. It can be modified with various functional groups to meet the requirements of drug carriers in different environments. Chitosan exhibits excellent mucosal adhesion and pH sensitivity, facilitating prolonged retention on mucosal surfaces and enhancing paracellular transport of therapeutic drugs [92]. The structure of CS not only possesses significant physicochemical properties but also exhibits specific interactions with cells and biomolecules [93]. Mostafa Set al. employed chitosan nanoparticles (CS-NPs) for in vivo delivery of anti-transforming growth factor-β1 (*TGF-β1*) siRNA, aiming to explore novel therapeutic strategies for liver fibrosis by downregulating *TGF-β1* expression in activated hepatic stellate cells (aHSCs). The study employed platelet-derived growth factor receptor-β (PDGF-β) binding peptides of varying densities to perform targeted modification of CS-NPs. The characterization results indicate that the modified nanoparticles exhibit an average hydrodynamic diameter of 103 ± 7 nm, a zeta potential of 24 ± 1 mV, and an siRNA encapsulation efficiency of 92.39 ± 6.4%. In the therapeutic application of a carbon tetrachloride (CCl_4_)-induced liver fibrosis mouse model, CS-NPs loaded with anti-*TGF-β1* siRNA and modified with high-density PDGF-β binding peptides significantly reduced *TGF-β1* and fibronectin levels in liver tissue by 65% and 63%, respectively. This formulation reduces inflammatory cell infiltration in the liver region, inhibits the proliferation of hepatic fibroblasts, and simultaneously decreases collagen deposition in the liver, offering a viable therapeutic approach for liver fibrosis [94]. Another researcher has developed an injectable thermosensitive chitosan/serine (CS/SS) hydrogel. This carrier, loaded with siRNA nanoparticles targeting cancer survival-associated viruses, holds promise for breast cancer treatment [95]. Furthermore, Yan L et al. developed a novel folate-targeted PLGA-PEG-stabilized chitosan–albumin nanocomplex (FA-PPC-siRNA) for efficient siRNA delivery to cancer cells. Characterization data indicate that FA-PPC-siRNA exhibits an average particle size of 186.62 ± 8.93 nm, a zeta potential of 23.61 ± 3.93 mV, and a high siRNA encapsulation efficiency of 96.28 ± 4.5%. In vitro release experiments demonstrated that the complex exhibited a cumulative siRNA release rate of 93.62 ± 3.48% within 24 h, with pH-dependent release characteristics. Functional validation results showed that FA-PPC-siRNA significantly inhibited lung cancer cell proliferation while exhibiting excellent biocompatibility and tumor suppression activity in the in vivo experiments [96].

Alginic acid is an anionic polysaccharide extracted from brown algae, primarily composed of glucuronic acid and mannuronic acid residues. Alginic acid exhibits excellent biocompatibility, biodegradability, and hydrophilicity. Researchers modified alginate to impart excellent biodegradability and biocompatibility without inducing immunogenicity [97]. Alginate, as an excellent material for creating matrices, can be modified to incorporate drugs, allowing them to be released at a specific rate to achieve drug delivery and localized treatment [98]. Alginate also enhances the diffusion rate of cationic siRNA in the static mucus barrier [99]. Ting L et al. investigated the effects of the *VEGF-C*/*VEGFR-3* (vascular endothelial growth factor receptor-3) signaling pathway on endothelial progenitor cell (EPC) differentiation. This study demonstrated that PEI-alginate nanoparticles delivering *VEGFR-3* siRNA inhibit lymphatic vessel formation in EPCs, thereby suppressing lymphatic metastasis of tumor cells. The size and surface charge of PEI-alginate nanoparticles carrying *VEGFR-3* siRNA (N/P = 16) were 139.1 nm and 7.56 mV, respectively. *VEGFR-3* siRNA specifically suppressed intracellular *VEGFR-3* mRNA expression. Following treatment with PEI alginate/siRNA nanocomplexes, EPCs failed to differentiate into lymphatic endothelial cells, and the proliferation, migration, and lymphangiogenesis of EPC-derived cells were significantly inhibited. This study provides a potential therapeutic approach for inhibiting tumor lymphangiogenesis and lymphatic metastasis [100]. Fernandez-Alarcon et al. have developed a metal–alginate (ALG) hydrogel containing high concentrations of metal ions such as Ca^2+^, combined with Zn^2+^, Li^+^, or Mg^2+^. This system can disrupt Ca^2+^ homeostasis in cancer cell mitochondria via local hyperthermia. Meanwhile, they loaded oncogene-silencing nucleic acids (mTOR siRNA) into polymer nanoparticles (NPs) composed of poly(β-amino ester), and embedded these nanoparticles into the metal–alginate hydrogel to target melanoma cells. The study revealed that the hydrogel can block glucose catalysis and affect the Tricarboxylic Acid Cycle (TCA Cycle), pentose phosphate pathway, and mitochondrial transport. By disrupting mitochondria in cancer cells, the ALG hydrogel activates the caspase immune cascade, thereby eliminating cancer cells and preventing tumor recurrence [101].

#### 3.2.5. Dendritic Polymers and Hyperbranched Polymers

Dendritic molecules and hyperbranched polymers represent a unique class of highly branched, nanoscale macromolecules that are characterized by three-dimensional tree-like structures that confer exceptional structural precision, surface functionality, and internal payload capacity [102]. The dendritic structure is a globular arrangement of multi-branched polymers, featuring a central core, repeating unit branches, and an outer layer of polyvalent functional groups [103]. These functional groups can form electrostatic interactions with siRNA, while the hydrophobic interior cavity can encapsulate uncharged, nonpolar molecules through multiple interactions. This structure simultaneously achieves multivalent surface conjugation of the target ligand, imaging agent, and stimulus-responsive group [104]. Branched molecules undergo multigenerational evolution, with polyamide-polyamide (PAMAM) and polypropyleneimine (PPI) having been employed for siRNA delivery. Padnya P et al. demonstrated for the first time that symmetrical polyamide dendrimers (PAMAM-calix-dendrimers) can form stable, positively charged complexes with siRNA, protecting them from enzymatic degradation and effectively delivering genetic material into HeLa cells. As the number of generations increases, PAMAM-calix-dendrimers exhibit a significant reduction in their toxicity to blood and cells, ultimately rendering these compounds non-toxic at concentrations required for siRNA binding and delivery [105]. Other researchers have constructed a class of ferrocene-based amphiphilic dendrimers (Fc-Am Ds). This complex exhibits significant ROS-responsive properties, enabling it to specifically disassemble ROS–siRNA complexes within ROS-rich cancer cells. This facilitates efficient siRNA delivery and gene silencing [106]. Additionally, dendritic molecules also exhibit a certain degree of pH sensitivity. Research employing all-atom molecular dynamics simulations investigated the complexation behavior of galactose-functionalized Gal-PAMAM and poly-Gal-PETIM dendrimers under two distinct pH conditions of 7 and 10. Researchers found that Gal-PAMAM/siRNA exhibits stronger binding affinity at pH 7, with greater structural fluctuations in siRNA within the Gal-PAMAM/siRNA complex compared to the Gal-PETIM/siRNA complex. However, in polar solvents, Gal-PETIM demonstrates superior hydrophobic properties, enabling rapid gene delivery under physiological pH conditions [107]. This study provides a valuable reference for the application of galactose-functionalized dendrimers under two different pH conditions.

### 3.3. Inorganic Nanoparticles

Inorganic nanoparticle delivery systems primarily load siRNA through electrostatic adsorption, pore-channel loading, and surface covalent bonding. Drug loading efficiency typically reaches 70–95%, but the loading capacity is significantly influenced by particle size, specific surface area, pore volume, and surface potential. The release kinetics primarily depend on pH response and ion exchange, exhibiting overall slow, sustained, and well-controlled characteristics. Approximately 10–30% is released within 24 h, with cumulative release typically reaching 40–70% by 72 h. Long-acting systems can maintain sustained release for several days to weeks. However, due to poor biodegradability and slow metabolism, incomplete release and long-term accumulation frequently occur in vitro and in vivo, with complete release rates that are generally below 80% [108].

#### 3.3.1. Gold, Silicon, and Calcium Phosphate Nanoparticles

Gold nanoparticles (AuNPs) possess specific physical and chemical properties, featuring a stable core and excellent biocompatibility, making them suitable for siRNA delivery. The direct binding method between siRNA and AuNPs involves attachment to the nanoparticle surface via a thiol-gold covalent bond. AuNPs enter monolayer fibroblasts via macropinocytosis and enter cells through clathrin-mediated endocytosis [109]. Research has demonstrated that *microRNA 21* (*miR-21*) suppresses the expression of *glucose transporter-1* (*Glut1*) in cancer cells by triggering a toehold-mediated strand displacement reaction. The glucose oxidase activity of these AuNPs accelerates intracellular glucose consumption, promoting cancer cell starvation. In combination with ROS signaling, this inhibits cancer cell proliferation and xenograft tumor growth while promoting apoptosis [110]. AuNPs can also be triggered by visible light irradiation to generate mild radiant heat, thereby enhancing their siRNA-loaded gene-silencing capability [111]. Georges M et al. conjugated AuNPs with dendrigraft Poly-L-Lysine (d-PLL), PEG-modified thiol PEG (SH-PEG-OCH) (molecular weight 5400 g/mol), and folic acid-modified thiol PEG (SH-PEG-FA) of similar molecular weight. This composite demonstrated certain applicability in prostate cancer therapy [112]. Elnaz S et al. developed shell-algae-coated AuNPs to protect siRNA. This composite LBL-CS-AuNPs-siRNA was transfected into H1299-eGFP lung epithelial cells, protecting siRNA from enzymatic degradation and serum instability while inducing endosomal escape to facilitate siRNA release at target sites [113]. These nanoparticles efficiently downregulate gene expression and demonstrate improved stability and therapeutic efficacy, with no significant cytotoxicity.

Mesoporous silicon nanoparticles (MSNs) possess a highly ordered pore structure and large surface area, enabling efficient drug loading and controlled release through covalent or non-covalent interactions with siRNA [114]. Shiuan et al. developed a topical formulation for transdermal siRNA delivery based on mesoporous silica nanoparticles (MSNPs), with a drug loading capacity of 1.4 μg of oligonucleotide per milligram of MSNPs. This siRNA was used to target *TGFβR-1* for the treatment of squamous cell carcinoma (SCC) of the skin. Compared to the control, MSNPs containing *TGFβR-1* siRNA demonstrated a twofold greater inhibitory capacity against *TGFβR-1* [115]. Another study developed a novel multifunctional nanodevice featuring a “core-shell” structure. This structure utilizes Fe_3_O_4_@SiO_2_ mesoporous silicon nanoparticles (MSNPs) as the core, which are then coated with PEI and modified with zwitterionic 2-methacryloyloxyethyl phosphorylcholine (MPC). This system exhibits excellent anti-protein adsorption properties in solutions containing BSA and FBS plasma proteins, enabling the co-delivery of siRNA and doxorubicin, with drug release regulated by an external oscillating magnetic field (OMF). Experiments confirmed that the loaded siGFP can silence GFP expression in Ovcar8 ovarian cancer cells. Following the co-release of anti-TWIST siRNA and doxorubicin, cytotoxicity against Ovcar8 cells increased by 50%. This system represents the first instance of utilizing zwitterionic “core-shell” nanoparticles to achieve synergistic delivery of anti-*TWIST* siRNA and daunorubicin for gene silencing, offering new possibilities for ovarian cancer treatment [116].

Calcium phosphate, owing to its excellent biocompatibility and stability, is also widely used in drug delivery systems. Researchers have employed calcium phosphate nanoparticles (CaPNPs) for siRNA delivery. However, due to poor endosomal escape under physiological conditions, the use of CaPNPs for siRNA delivery presents certain limitations [117]. Fei M Y et al. enhanced the siRNA delivery efficiency of CaPNPs by modifying their surface with the cell-penetrating peptide (TAT) and preparing the Mg-CaPNPs-RGD-TAT-*CKIP-1* siRNA carrier system through hydrothermal synthesis, silanization, and adsorption [118].

#### 3.3.2. Magnetic Nanoparticles

Superparamagnetic iron oxide nanoparticles (SPIONs) are widely used as contrast agents in magnetic resonance imaging (MRI) and have been approved for liver imaging to detect lesions or fibrotic changes. SPIONs serve as drug delivery systems due to their unique properties. Their large surface area enables binding to siRNA and other targeting ligands, facilitating targeted therapy. Dandan S et al. developed glycyrrhetinic acid-modified lipid-poly(aspartic acid)-poly(ethyleneimine) superparamagnetic nanoparticles (GLPPS) as a visualizable liver-targeted siRNA carrier to enhance siRNA delivery efficiency and diagnostic capabilities in hepatocellular carcinoma. Research indicates that GLPPS exhibits significant targeting specificity and excellent biocompatibility in vitro and in vivo with no apparent toxicity, and simultaneously reduces tumor MRI signal intensity while enhancing imaging contrast. It represents a highly promising liver-targeted siRNA delivery and MRI monitoring vector [119]. Goknur K et al. creatively employed sericin (Ser) as a coating material to prepare SPIONs, which were then modified with poly-L-lysine (PLL) to encapsulate negatively charged siRNA. Characterization revealed spherical nanoparticles with a narrow size distribution. Ser coating formed a characteristic layered structure, while PLL modification did not significantly alter particle size but imparted a positive charge while retaining superparamagnetism. siRNA binding efficiency reached 81.90–93.50%. This complex exhibits good biocompatibility with both cancerous and non-cancerous cells, and does not inhibit cell colony formation except at extremely high doses [120]. Song Y constructed PEG-PCL-PEI-C14-SPIONs (PPPCSs) by combining PEI and PCL-based SPIONs with PEG-modified surface structures. These nanoparticles suppress the overexpression of circ_0058051, thereby addressing the poor prognosis in hepatocellular carcinoma (HCC) patients. PPPCSs protect circ_058051 siRNA from degradation in serum and efficiently deliver it into SMMC-7721 cells, demonstrating tumor growth inhibition in a subcutaneous tumor model [121]. These experiments confirm SPIONs’ significant potential as a novel and promising method for siRNA delivery.

### 3.4. Protein Nanoparticles

Protein-based nanoparticles (PNPs) serve as alternative carriers for synthetic nanoparticles and are widely utilized in nanoparticle-based drug delivery systems. Proteins can bind to and carry large quantities of drugs through various mechanisms, including electrostatic interactions, hydrophobic interactions, and covalent bonds. However, drug loading efficiency is easily affected by the protein structure and preparation conditions. Based on the tertiary structure of proteins, nanoparticles with varying surface areas can be designed, thereby influencing the interaction between the carrier and target cells [122]. Protein nanoparticles primarily load siRNA through self-assembled cavity encapsulation, electrostatic complexation, or hydrophobic interactions. Drug loading efficiency typically ranges from 60% to 90%, and is significantly influenced by protein type, assembly conditions, and the N/P ratio. Release kinetics primarily involve protein degradation, disassembly, or microenvironmental responses, exhibiting low burst release and slow, controlled characteristics. Approximately 15–35% is released within 24 h, with cumulative release typically reaching 50–80% between 48 and 72 h. Release can be significantly accelerated under protease, pH, or reducing conditions, though the overall rate remains moderate with considerable variability [123]. Proteins also exhibit excellent biocompatibility, minimizing immune reactions and toxicity to the greatest extent possible, making them a crucial area of research in nanomedicine [124]. Han et al. developed a novel siRNA delivery system utilizing cationic bovine serum albumin (cBSA) nanoparticles with a controllable charge density. This system demonstrated highly efficient gene-silencing effects, inducing significant cancer cell apoptosis and thereby inhibiting tumor growth in a B16 lung metastasis model [125]. Aditi M er al. employed a solvation-free method to prepare bovine serum albumin (BSA) nanoparticles that target the *KRAS G12S* gene—the most frequently mutated gene in human cancers. In the *KRAS G12S* mutant A459 lung adenocarcinoma cell line, this PNP effectively encapsulates and protects siRNA payloads, enabling their in vitro delivery to A549 cells. It evades endogenous encapsulation and mediates significant sequence-specific *KRAS* silencing, resulting in slowed growth of siRNA-transfected lung cancer cells [126]. Choi et al. constructed siRNA capsid nanocarrier complexes that effectively suppressed RFP gene expression in the in vitro cellular models. The protective encapsulation of siRNA by the lipid shell shields it from degradation by plasma nucleases, prolonging its in vivo half-life. Concurrently, the multivalent RGD peptide on the carrier surface enables efficient tumor delivery through targeted binding to tumor cell integrin receptors. Combined with its extended circulation properties in vivo, this dual mechanism ensures highly effective in vivo RNA interference [127]. PNP has proven to be an effective approach for rationally designing vaccines, particularly those targeting complex pathogens and emerging infectious diseases. Multiple studies targeting SARS-CoV-2’s PNP have been conducted, revealing that PNP is highly suitable for multivalent antigen presentation and enhanced immune stimulation to induce potent humoral and cellular immune responses, demonstrating strong development potential [128,129].

### 3.5. Hybrid Nanoparticles

Hybrid nanocarriers, particularly lipid–polymer hybrids (LPHNs) and exosome-mimetic systems, represent more advanced platforms. By combining different delivery methods to achieve synergistic effects, it demonstrates enhanced biocompatibility and gene-silencing capability. Hybrid nanoparticle delivery systems typically load siRNA through core–shell structures, electrostatic complexation, and hydrophobic assembly. Drug loading efficiency generally reaches 70–95%, with a loading capacity that is highly correlated to component ratios and charge ratios. Batch stability outperforms that of single inorganic or protein carriers. Release kinetics typically exhibit a biphasic pattern: an initial mild burst release (10–30%) within the first 24 h, followed by a slow sustained release phase where cumulative release over 48–72 h generally reaches 50–85%. Triggered accelerated release can be achieved under stimuli such as pH, GSH, or enzymes, with the release rate quantitatively correlated to material degradation and bond-breaking rates. Lipid–polymer hybrid nanoparticles (LPHNs) feature a core composed of biodegradable polymers such as PLGA, PCL, or CS, encapsulating therapeutic agents, with an outer layer consisting of a lipid shell formed by lecithin, DSPE-PEG, or cholesterol. This lipid shell mimics the structural and functional characteristics of biological membranes, enhancing the biocompatibility of LPHNs while offering superior mechanical stability and controlled drug release. It also facilitates enhanced cellular uptake efficiency through lipid interface mediation [130]. Further functionalization with targeted ligands such as GalNAc, folate, or glycyrrhizic acid can enhance receptor-mediated endocytosis and organ-specific targeting [131]. Representative delivery systems include PLGA-PEG conjugated with lecithin for liver cell targeting, CS-lipid hybrids for HCC treatment, and folic acid-conjugated lipopolymer hybrids for drug delivery targeting HepG2 cells. Researchers have developed antibody-functionalized hybrid phospholipid–polymer nanoparticles for siRNA delivery, targeting receptors such as *EGFR* and *TROP2* that are overexpressed in triple-negative breast cancer (TNBC). These nanoparticles simultaneously carry multiple siRNAs to reduce off-target risks, demonstrating the complex’s potential as a potent and safe therapeutic approach [132]. Cai Y et al. developed a novel nanoparticle formulation, MVNP-CA/IFN-γ, featuring a poly(lactic-co-glycolic acid) (PLGA) core encapsulated within a dual-functional lipid shell composed of mannitol (MAN) and vancomycin (VAN). This formulation enables the co-delivery of the antibiotic chlortetracycline A (CA) and the immune activator interferon-γ (IFN-γ) for targeted clearance of intracellular methicillin-resistant Staphylococcus aureus (MRSA). Research confirms that MVNP-CA/IFN-γ nanoparticles exert a cascade-targeted effect against MRSA within macrophages. The core mechanism involves CA directly eliminating MRSA-resistant strains, while IFN-γ reprograms macrophages from the M2 phenotype to the M1 phenotype, thereby enhancing immune clearance capacity. This dual-action strategy efficiently eradicates MRSA-resistant bacterial populations, effectively addressing bacterial tolerance and immune evasion challenges, and offers a highly promising novel therapeutic approach for chronic MRSA infections [133].

Exosome-mimetic nanocarriers, inspired by natural extracellular vesicles (EVs), are engineered to mimic the physicochemical properties, endogenous targeting capabilities, and biological communication mechanisms of native exosomes [134]. These bionic systems exhibit high stability and low immunogenicity in systemic circulation. Their intrinsic cellular uptake mechanisms—such as membrane fusion, endocytosis, and pinocytosis—enable the efficient delivery of RNA therapeutics, small molecules, or proteins directly to target cells. Directly transfected exosomes can deliver small RNAs to podocytes in vitro, suggesting their potential as RNA carriers for therapeutic strategies in more complex environments [135]. Perera R et al. developed B7-H3-CAR-T exosomes capable of crossing the blood–brain barrier (BBB), targeting tumor cells expressing the B7-H3 protein, and delivering therapeutic siRNA to silence *HLX* expression in glioblastoma multiforme (GBM). CAR-T exosomes can induce tumor cell death through perforin and granzyme B content, demonstrating a potent inhibitory effect and the ability to promote cell apoptosis, while having lower cytotoxicity [136]. Lian B et al. established an exosome-based si-*LINC02544* system to silence *LINC02544*, thereby reducing *CAPRIN1* levels, upregulating *miR-497-5p*, and inhibiting the proliferation and migration of TNBC cells. This approach offers a promising therapeutic strategy for overcoming immunotherapy resistance in TNBC [137].

### 3.6. Coupling Technology

Conjugation technologies aim to achieve precise delivery of siRNA to extrahepatic target tissues. The conjugated delivery system directly links siRNA to targeting/delivery molecules via chemical bonds, achieving nearly 100% loading efficiency, precise drug loading, high batch-to-batch consistency, and eliminating issues with free drug residues. Release mechanisms depend on chemical bond cleavage, enzymatic degradation, or microenvironmental responses, enabling stable, controllable, and highly time-delayed release processes that achieve intracellular-specific activation and release. In vitro, it typically exhibits a slow release over 24–48 h, with complete release gradually achieved within 48–72 h. While intracellular activation efficiency is high, the complete release rate in vitro is generally below 80%. Furthermore, the release rate is highly dependent on the type of chemical bond and the intensity of microenvironmental stimulation [138]. These conjugates include cell-penetrating peptide (CPP)–siRNA conjugates, antibody–siRNA conjugates (ARC), and nucleic acid aptamer–siRNA conjugates.

CPPs are short peptides consisting of fewer than 30 amino acids that can deliver various substances into cells through mechanisms such as cell membrane perforation. The commonly used CPP is the TAT transcription activator derived from HIV-1. CPPs enhance siRNA transfection efficiency but also enter normal cells, causing off-target toxicity [139]. Some researchers have inserted a redox-cleavable linker between the PI and cRGD moieties. This design maintains low toxicity and targeting capability while enabling disulfide bond cleavage via thiol-disulfide exchange on the cell surface. This activates the CPP, promotes membrane translocation, and ultimately achieves efficient cytoplasmic siRNA delivery [140]. Wan Y successfully synthesized a unique structure formed by peptide modification and fluorination (DEN-TAT-PFC). This peptide consists of dendritic polylysine, the cell-penetrating peptide TAT, and perfluorocarbon (PFC), designed to load small interfering RNA targeting hypoxia-inducible factor-1α (si*HIF-1α*) and sorafenib(SF). This delivery system exhibits superior oxygen-carrying capacity and significantly enhances siRNA delivery efficiency. It effectively suppresses *HIF-1α* expression at both the RNA and protein levels, thereby alleviating hypoxia in tumor tissues and further downregulating vascular endothelial growth factor (*VEGF*) expression [141].

Antibodies induce a series of immune responses by binding to specific receptors (antigens) on the surface of target cells, ultimately leading to the degradation and death of these cells. Leveraging the specificity and targeting capabilities of antibodies, ARC has emerged as an efficient targeted delivery system for precisely delivering siRNA to specific target cells or tissues. Ren Q et al. developed a photosensitizer nanoparticle composed of Fe_3_O_4_@ZnO co-loaded with anti-*EGFR* antibody, brucella, and *Nrf2*-siRNA to enhance the therapeutic efficacy of photodynamic therapy (PDT). Following treatment with this photosensitizer, ROS levels in skin squamous cell carcinoma cells increased by 191.09% ± 10.02% through the inhibition of *Nrf2* and its associated antioxidant defense mechanisms, significantly enhancing the cytotoxic effect. This photosensitizer achieved a tumor inhibition rate of 76.30 ± 5.12% in the nude mouse model, demonstrating potent antitumor efficacy and enabling combination therapy with chemotherapy, gene therapy, and photodynamic therapy [142]. Dang N M et al. developed therapeutic nanoparticles (NPs) with *FZD7* antibody and β-chain protein small interfering RNA (siRNA) functions. The *FZD7* antibody specifically binds to and inhibits Wnt pathway signaling in TNBC cells by trapping the *FZD7* receptor in a ligand-unbound state, while siRNA more effectively suppresses cell proliferation, migration, and spheroid formation by inhibiting β-catenin through RNA interference [143]. Wang et al. developed a novel photo-responsive antibody-siRNA conjugate (PARC), designed to enable remote, controlled siRNA delivery, offering an innovative technical solution for tumor immunogene therapy. This PARC covalently conjugates the *PD-L1* antibody with small interfering RNA (si*PD-L1*) targeting *PD-L1* mRNA via a photolabile o-nitrophenyl group. During treatment, light stimulation can be applied at predetermined time points to trigger the cleavage of the photolabile bridge in the PARC molecule. This releases si*PD-L1* from the conjugate, allowing it to enter the cytoplasm and subsequently degrade intracellular *PD-L1* mRNA in a specific manner. Simultaneously, the *PD-L1* antibody (α*PD-L1*) within the conjugate binds to *PD-L1* molecules on the cell membrane surface, blocking their function and effectively enhancing the activity of immune cells. This study not only validated the feasibility of the light-responsive antibody–RNA interference (siRNA) conjugate (ARC) system for siRNA-targeted delivery but also provided novel strategies and technical approaches that combine precision and controllability for the field of tumor immunotherapy [144].

Nucleic acid aptamers can recognize target molecules through their three-dimensional conformation and are more readily coupled via base complementary pairing or covalent bonding. Nucleic acid aptamers range in length from 20 to 100 nucleotides, possess a relatively low molecular weight, exhibit reduced immunogenicity and toxicity, and demonstrate strong tissue permeability, making them advantageous for drug delivery. Zhao L et al. constructed a DNA soccerball framework (DSF) delivery vehicle by synergistically functionalizing SGC8 aptamers with fluorocarbon chains. Grafting fluorocarbon groups of controllable length and density onto the DSF surface promotes phagocytosis, evades lysosomal degradation, and enhances siRNA transport efficiency into the cytoplasm. Among the 90 available modification sites on DSF, fluorocarbon groups exhibit synergistic enhancement effects with sgc8 aptamers when surface-modified at a 3:1 ratio, significantly amplifying siRNA-mediated gene silencing by improving both internalization efficiency and cytoplasmic release efficacy. When siRNA targeting the *Bcl2L12* gene was loaded, this optimized DSF variant induced the highest levels of apoptosis in both cancer cell lines while maintaining extremely low cytotoxicity, demonstrating excellent biosafety and therapeutic specificity [145]. Kwak M et al. developed an AS1411 aptamer-functionalized liposome platform encapsulating siRNA targeting metastasis-associated protein 2 (*MTA2*). AS1411-Lipm inhibits the tumor suppressor protein *PTEN* and activates the PI3K/AKT signaling pathway, demonstrating high siRNA encapsulation efficiency, selective uptake in radionuclide-positive PDAC cells, and enhanced endosomal escape capability [146]. Ning H et al. developed a novel nucleic acid drug delivery system, AS1411/LNP, targeting the *circPDHK1* circRNA to inhibit the proliferation and metastasis of clear cell renal cell carcinoma (ccRCC). The system successfully inhibited phosphorylation of the mTOR-AKT pathway, suppressed proliferation and migration of ccRCC cells, and exhibited minimal side effects in vital organs [147].

## 4. siRNA Drugs for Cancer Treatment Currently in Clinical Research

To date, numerous siRNA-based therapies remain in clinical research [148] (Table 4). ALN-VSP02 is indicated for solid tumors, targeting *VEGF* and *KSP*, administered via systemic intravenous infusion, and has completed Phase I clinical trials. siRNA-*EphA2*-DOPC for advanced cancer is administered via systemic intravenous infusion, targeting *EphA2*. Its Phase I clinical trial is ongoing. Atu027 is indicated for advanced or metastatic pancreatic cancer (Phase II) and solid tumors (Phase I), targeting *PKN3*. Administered via systemic intravenous infusion, it has completed Phase II clinical trials. TKM-*PLK1* (TKM-080301) targets *PLK-1* for adrenal cortical carcinoma (Phase II), hepatocellular carcinoma (Phase II), neuroendocrine tumors (Phase II), and solid tumors (Phase I). Administered via systemic intravenous infusion, it has completed Phase II clinical trials. siG12D LODER is indicated for pancreatic ductal adenocarcinoma and pancreatic cancer, targeting the *KRAS G12D* mutation. It is administered via local surgical implantation and is currently in Phase II clinical trials, which are ongoing.

DCR-*MYC* was the first LNP-formulated siRNA targeting *MYC* to enter clinical testing. In two Phase I trials (DCR-*MYC*-101 and DCR-*MYC*-102), the agent demonstrated acceptable safety and proof of mechanism with confirmed MYC mRNA knockdown in tumor biopsies. However, the magnitude of MYC suppression was insufficient to induce meaningful clinical responses, and no objective antitumor activity was observed in hepatocellular carcinoma or other solid tumors. Dicerna formally discontinued clinical development in September 2016. Additionally, a case study reported that a 70-year-old female patient developed renal thrombotic microangiopathy (TMA) after receiving monotherapy with a short-interfering RNA (DCR-*MYC*) targeting MYC for adenoid cystic carcinoma of the breast. Clinical and pathological changes in patients are closely associated with the initiation and subsequent discontinuation of DCR-*MYC* therapy. A gradual decline in renal function was observed after several months of drug administration, and partial reversal of clinical and laboratory abnormalities occurred following cessation of DCR-*MYC* treatment. Researchers concluded that the most probable cause of the patient’s progressive renal failure was anti-*MYC* miRNA therapy [149]. This finding provides crucial insights into the potential nephrotoxicity of novel targeted therapies, offering important implications for the clinical management of cancer patients receiving this or similar treatments in the future.

**Table 4:** siRNA preparations currently in clinical trial research phases.

Therapeutic Drugs and Clinical Trial Identifiers	R&D Institutions	Target Gene/Protein	Indications	Nanocarriers	Phase/Status
NU-0129 [150] (NCT03020017)	Northwestern University	*Bcl2L12*	Glioblastoma, recurrent glioblastoma	Gold nanoparticles	Phase I (Completed)
Mesenchymal stem cell-derived exosomes loaded with *KRAS G12D*-siRNA [151] (NCT03608631)	M.D. Anderson Cancer Center	*KRAS G12D*	Metastatic pancreatic cancer, pancreatic ductal adenocarcinoma, stage IV pancreatic cancer	Exosomes	Phase I (Recruiting)
TKM-080301 [152] (NCT02191878)	National Cancer Institute	*PLK1*	Colorectal cancer with liver metastases, pancreatic cancer, gastric cancer, breast cancer, ovarian cancer	Lipid Nanoparticles	Phase I (Completed)
NBF-006 (NCT03819387)	Nitto BioPharma, Inc.	*GSTP1*	Non-small cell lung cancer, pancreatic cancer, colorectal cancer	Lipid Nanoparticles	Phase I (Completed)
siG12D SOLDER [153] (NCT01676259)	Silenseed Ltd.	*KRAS G12D*	Pancreatic ductal adenocarcinoma, pancreatic cancer	Polymer Nanoparticles	Phase I (Completed)
DOPC-encapsulated siRNA targeting *EphA2* (NCT01591356)	M.D. Anderson Cancer Center	Epidermal growth factor-like domain 2	Advanced malignant solid tumors	Neutral lipid nanoparticles	Phase I (Recruiting)
DCR-*MYC* (NCT02110563)	Digested Pharmaceuticals, Inc.	*MYC*	Liver cancer	Lipid Nanoparticles	Phase I/II (Discontinued)
Go to 2 (NCT06795815)	Silence Therapeutics	*PKN3*	Advanced or metastatic pancreatic cancer(II), solid tumors(I)	Lipid Nanoparticles	Phase II/Completed
ALN-VSP02 (NCT00882180)	Alnylam Pharmaceuticals	*VEGF, KSP*	Solid tumors	Nucleic acid lipid particles (SNALP)	Phase I/Completed
CALAA-01 [18] (NCT00689065)	Calando Pharmaceuticals	*RRM 2*	Solid tumors	cyclodextrin-based nanoparticles	Phase I/Terminated

Data Source: ClinicalTrials.gov.

### 4.1. Atu027 (Clinical Trial Identifiers, NCT01808638)

Atu027 is a siRNA-based lipid nanoparticle capable of knocking down protein kinase N3 (*PKN3*) expression. Elevated *PKN3* expression correlates with enhanced angiogenesis and tumor metastasis. Therefore, silencing *PKN3* expression may inhibit angiogenesis and tumor invasion. Beate S et al. initiated a clinical trial to evaluate the safety of Atu027 in combination with gemcitabine in patients with advanced pancreatic cancer (APC), and assessed its pharmacokinetics and efficacy. A total of 23 patients with unresectable metastatic pancreatic adenocarcinoma were enrolled and randomly assigned to receive gemcitabine combined with two different Atu027 dosing regimens: 0.235 mg/kg once weekly and 0.235 mg/kg twice weekly. During treatment, disease control rates were achieved in 4/11 patients in Cohort 1 and 7/12 patients in Cohort 2. Patients in Cohort 1 maintained a stable overall health status, while those in Cohort 2 demonstrated marked improvement in overall health status. Atu027 demonstrated favorable safety and tolerability when combined with gemcitabine, with patients receiving twice-weekly dosing. Atu027 was associated with significantly improved prognosis [154]. The findings of this clinical study substantiate the pivotal role of vascular endothelium in tumor metastasis, providing robust support for subsequent targeted therapy research on this pathway. Further in-depth exploration is urgently needed.

### 4.2. SIG12D LODER (Clinical Trial Identifiers, NCT01676259)

siG12D LODER is the first intratumoral siRNA nanotherapeutic for cancer treatment, having received clinical evaluation in 2011. siG12D LODER is a sustained-release, long-acting local delivery system composed of LOcal Drug EluteR copolymer (LODER) and embedded anti-*KRAS* siRNA (siG12D). In 2020, A Phase II clinical trial for siG12D-LODER commenced, aiming to compare the efficacy of gemcitabine plus napacirolal versus gemcitabine plus siG12D-LODER in treating locally advanced pancreatic cancer(LAPC) [155]. Ultimately, the clinical trial enrolled a total of 59 patients, who were divided into two cohorts. In cohort 1, patients were randomly assigned to receive siG12D-LODER plus gemcitabine/albumin-bound paclitaxel (Group 1) or gemcitabine/albumin-bound paclitaxel alone (Group 2). In cohort 2, patients with locally advanced or borderline resectable disease received siG12D-LODER combined with standard chemotherapy (modified FOLFIRINOX regimen or gemcitabine/albumin-bound paclitaxel). Primary endpoints were overall survival (OS) for cohort 1 and objective response rate (ORR) for cohort 2. In cohort 1, the median OS in the modified intention-to-treat (mITT) population without *KRAS* status screening was 7 months for group 1 and 21.9 months for group 2. Among patients with *KRAS G12D/V* mutations, OS was 22.7 months and 13.4 months, respectively. In cohort 2, ORR was 31.6% in the mITT population and reached 57.1% in the *KRAS G12D/V* subgroup. This study evaluated the efficacy and safety of siG12D-LODER combined with chemotherapy in LAPC [153]. The results indicate that siG12D-LODER combined with chemotherapy demonstrates superior therapeutic efficacy in *KRAS G12D/V* patients, with high drug safety. This warrants further investigation in LAPC harboring *KRAS G12D/V* mutations.

Notably, in ongoing siRNA therapy clinical trials, approximately 70% utilize lipid nanoparticles (LNPs) as delivery vehicles, while other nanocarrier systems rarely advance to late-stage clinical development. This may be attributed to the significant advantages of LNPs. LNPs exhibit efficient cellular uptake and pH-triggered endosomal escape, ensuring effective siRNA release into the cytoplasm. LNPs exhibit well-defined toxicity profiles, low immunogenicity, and predictable pharmacokinetic behavior [156]. Moreover, multiple LNP-based siRNA therapeutics have received regulatory approval, providing a clear translational pathway. In contrast, other types of nanocarriers face significant clinical barriers. Inorganic nanoparticles exhibit poor biodegradability, pose potential risks of long-term tissue accumulation, and have unclear biosafety profiles. Traditional liposomes suffer from inadequate colloidal stability, unstable siRNA loading, and weak endosomal escape capabilities. Biomimetic or cell-derived carriers encounter challenges in batch-to-batch consistency, large-scale production, and regulatory approval. This provides research insights for developing siRNA nanocarriers. On one hand, we must optimize the biocompatibility, degradability, and endosomal escape efficiency of carriers at the molecular design level, reduce toxicity and immunogenicity caused by cationic materials, and narrow the gap between other nanocarriers and LNPs in terms of safety and delivery efficiency. On the other hand, establishing standardized preparation and quality control systems is essential to address the significant batch-to-batch variability and scalability challenges associated with inorganic, polymeric, and biomimetic carriers. Concurrently, intensifying research into in vivo pharmacokinetics and tissue distribution mechanisms will provide more robust clinical translation evidence for non-LNP carriers.

Additionally, numerous siRNA-based therapeutic agents targeting other diseases are currently in clinical and preclinical trial phases. These siRNA-based therapies have demonstrated promising outcomes across multiple conditions and offer the potential to address diseases at the genetic level. Other RNA therapeutics, including miRNAs and antisense oligonucleotides (ASOs), are also gaining increasing attention in clinical research.

## 5. Discussion

Theoretically, RNA-based therapies, particularly siRNA, hold potential as future therapeutic strategies. siRNA can treat any disease by silencing specific genes associated with disease progression through surface group modification. However, delivering siRNA requires overcoming extracellular barriers and intracellular barriers. These barriers can be overcome by chemically modifying siRNA or developing viable delivery systems. To date, various delivery systems have been developed, and multiple drugs have entered clinical trial phases. However, we still need to conduct practical in vivo efficacy and safety assessments to reduce cytotoxicity and immune activation. Future siRNA therapies will continue to expand into major diseases such as cancer and neurodegenerative disorders, demonstrating significant potential when combined with immunotherapy and chemotherapy regimens. However, challenges remain, including low delivery enrichment rates in extrahepatic tissues and the difficulty of overcoming physiological barriers like the blood–brain barrier. The GalNAc-conjugated platform has been approved for liver-specific gene silencing, which may address liver targeting issues. However, we still need to develop other nanoparticle siRNA therapeutics suitable for treating other diseases. All these studies indicate that there remains significant scope for developing effective nanoparticle systems. These nanoparticle systems need to efficiently target mRNA, enhance endosomal escape, and reduce off-target effects through binding to targeting ligands.

## Figures and Tables

**Table 1:** The preparation methods, advantages, and limitations of antibody, peptide, and aptamer modification methods.

Modification Methods	Preparation Method	Advantages	Limitations
Antibody modification [32]	Post-synthetic covalent conjugation Site-specific conjugation Non-covalent self-assembly Fusion protein strategy	High targeting specificity Strong affinity enables precise recognition of tumor cell surface antigens Extended circulation time of siRNA in vivo	High molecular weight Elevated immunogenicity risk Complex preparation and conjugation processes High costs Limited tissue penetration in vivo
Peptide modification [33]	Solid-phase co-synthesis Post-synthetic conjugation in solution Non-covalent self-assembly method	Low molecular weight Exceptional tissue permeability Simple synthesis methods Some peptides also possess membrane-penetrating capabilities, simultaneously enhancing targeting and intracellular delivery efficiency	Prone to protease degradation Poor stability in vivo Poor egression from the endosome
Aptamer modification [34]	Solid-phase co-synthesis Post-synthetic conjugation in solution Enzymatic ligation Non-covalent hybridization	Obtained through in vitro screening With broad target specificity Low molecular weight No significant immunogenicity Easy synthesis	Prone to degradation by nucleases in vivo requires chemical modification for stability Its affinity still falls short of that of antibodies

**Table 2:** Examples of cell membrane-coated nanoparticles for cancer.

Membrane Source (Cell Type)	Target-Locking Mechanism	Nanocarrier	Load Medication	Cancer Model	Therapeutic Effects
Tumor cells	Homotypic Interaction (tumor cell affinity)	SP-PLB NPs	Palbociclib	murine oral cancer cells (melanoma, mouse)	These nanoparticles enhance isotype cell uptake, reactive oxygen species (ROS) production, mitochondrial dysfunction, and potent cytotoxicity [42]
Macrophage	Tumor Endothelial Recognition	ZID@RM	DOX	Hepa1-6 Tumor Model	Synergistic therapy of PTT/PDT/chemotherapy inhibits tumor growth and metastasis [43]
Erythrocyte membranes	Tumor Recognition	Nanogel	*miR155*	BV-2 cells BV-2	Active tumor-targeting capability and excellent tumor inhibition efficacy [44]
Platelet membranes	Tumor Recognition	ZIF-8 MOF ZIF-8	Survivin siRNA	SK-BR-3 cells SK-BR-3	High silencing efficiency and significant antitumor targeting and therapeutic efficacy [45]
Stem cell membranes	Tumor Recognition	PDA	DOX and *PDL1*	PC-3 cells PC-3	Effectively enhanced blood retention and improved accumulation at tumor sites with synergistic chemoimmunotherapy [46]

**Table 3:** Polymeric micelles for siRNA delivery [77].

Micelle Composition	siRNA Dose	Target Gene/Protein	Potential Outcomes
PEG-SS-siRNA/PEI [78]	one hundred nanomolar	*VEGF*	These micelles encapsulated siRNA successfully transfected into the prostate carcinoma cells (PC-3) and silenced the *VEGF* gene expression up to 96.5%.
Lactose-PEG-siRNA/PLL [79]	one hundred nanomolar	luciferase	The micelle-based siRNA was delivered into the hepatoma cells, and up to a 100-fold increase in gene-silencing activity was observed.
6PEG-siRNA-Hph1/KALA [80]	75 pmol	GFP	This siRNA combination with micelles is effective in delivery and prevents enzymatic degradation. It inhibited the *GFP* gene expression in MDA-MB-435 cells.
LHRH-PEG-SS-siRNA/PEI [81]	50 nanomolar	*VEGF*	These micelles showed increased cellular uptake compared to those without LHRH and caused effective *VEGF* gene silencing.
PEG-SS-siRNA/PEI [82]	one hundred nanomolar	*VEGF*	These nanocarriers, delivered by the intratumoral route, silenced the *VEGF* expression without any inflammatory response in vivo.
PDOT-Ms/si*PLK1* [83]	50 nanomolar	*PLK1*	PDOT-Ms/si*PLK1* efficiently targeted *PLK1* gene expression in HepG2-xenograft highly malignant patient-derived xenograft models by promoting the release of siRNA into the cytosol.

## Data Availability

No new data were created or analyzed in this study. Data sharing is not applicable to this article.
